# Racial and Ethnic Disparities in Adverse Pregnancy Outcomes Among Women with Early Onset Cancer in the United States

**DOI:** 10.3390/cancers18071081

**Published:** 2026-03-26

**Authors:** Duke Appiah, Julie Sang, Eric K. Broni, Zheng Shi, Catherine Kim

**Affiliations:** 1 Department of Public Health, Julia Jones Matthews School of Population and Public Health, Texas Tech University Health Sciences Center, Lubbock, TX 79430, USA; 2 School of Medicine, Texas Tech University Health Sciences Center, Lubbock, TX 79430, USA; julie.sang@ttuhsc.edu; 3 Department of Obstetrics, Gynecology and Reproductive Sciences, School of Medicine, Yale University, New Haven, CT 06520, USA; eric.broni@yale.edu; 4 Radiation Oncology Clinic, University Medical Center Cancer Center, Lubbock, TX 79430, USA; zheng.shi@ttuhsc.edu; 5 Texas Tech University Health Sciences Center, Lubbock, TX 79430, USA; 6 Departments of Medicine and Obstetrics and Gynecology, University of Michigan, Ann Arbor, MI 48109, USA; cathkim@med.umich.edu

**Keywords:** adverse pregnancy outcomes, race, cancer, ethnicity, early onset cancer, epidemiology

## Abstract

Background: Despite well-established racial/ethnic disparities in cancer outcomes, little is known about the extent to which race/ethnicity influences adverse pregnancy outcomes (APOs) among women with early onset cancer. We evaluated racial/ethnic disparity in the occurrence of cancer during pregnancy and APOs among women with cancer in the United States. Methods: Data consisted of 17.6 million singleton deliveries among females aged 18–49 years from the National Inpatient Sample. Logistic regression models were used to estimate the odds ratios (ORs) and 95% confidence intervals (CIs). Results: From 2000 to 2022, the prevalence of births among women with cancer increased more than 225%, from 120.4 to 391.8 per 100,000. After accounting for sociodemographic and behavioral/lifestyle factors and comorbidity index among women with cancer (*n* = 49,824, mean age = 33.4 years), non-Hispanic Black women had the highest odds for hypertensive disorders of pregnancy (OR = 1.67, CI: 1.54–1.82), preterm birth (OR = 1.44, CI: 1.26–1.64) and fetal death (OR = 3.04, CI: 1.99–4.63). Asian or Pacific Islander and Native American women had the highest odds for gestational diabetes (OR = 2.48, CI: 2.17–2.85) and fetal growth restriction (OR = 1.92, CI: 1.00–3.69), respectively. Among racial/ethnic minority women, the odds for maternal mortality and several APOs were significantly higher among those with cancer than those without cancer, with the odds for APOs being highest for breast cancer (OR = 1.39, CI: 1.23–1.56). Conclusions: This large population-based study showed significant racial and ethnic disparities in APOs among women with a concurrent cancer diagnosis at delivery. Targeted management of APO risk factors during pregnancy among racial/ethnic minority populations with cancer may help reduce adverse maternal and neonatal outcomes.

## Simple Summary

Despite well-established racial and ethnic disparities in cancer outcomes, little is known about the extent to which race/ethnicity influences adverse pregnancy outcomes among women with early onset cancer. Using data of 17.6 million singleton pregnancy-related hospitalizations among female adults aged 18 to 49 years from the National Inpatient Sample (2000–2022), we observed that the prevalence of births among women with cancer increased by more than 225%. Furthermore, racial and ethnic minority women had a higher risk for adverse pregnancy outcomes compared to non-Hispanic White women with cancer. The findings of the current study hold significant clinical importance as they inform efforts to achieve maternal health equity in the United States.

## 1. Introduction

It is reported that about 1 in 1000 pregnancies occur among women diagnosed with cancer [1,2]. While the occurrence of cancer during pregnancy is an uncommon event, recent data show that the prevalence of concurrent cancer and pregnancy is on the rise [3,4]. About 88% of such pregnancies result in live births, with almost half of them being preterm births [5]. Drivers of the increasing trend in early onset cancer diagnosis, which is commonly defined as cancer diagnosis in adults occurring before age 50 years, during pregnancy, are largely unknown but may reflect the increasing prevalence of cancer among reproductive-age women [3,6].

Current estimates show that the prevalence of cancer at delivery increased from 3.41 per 10,000 deliveries in 2004 to 4.33 per 10,000 deliveries in 2013 in the United States [3]. While cross-sectional evaluations show a greater proportion of cases of cancer diagnosed during pregnancy occur among non-Hispanic White women [3], data on trends in the prevalence of concurrent cancer and pregnancy by race and ethnicity are not available, probably due to limited samples. With the rise in cancer among reproductive-age women over the last decade, surpassing that of men and racial and ethnic minority reproductive-age women experiencing varying degrees of cancer disparities [7], updated estimates of the prevalence of cancer among pregnant women are needed to characterize and address the unique medical needs of this significantly growing population.

Cancer during pregnancy poses important challenges in obstetric and oncologic care and management [8,9]. Mothers with cancer, as well as their offspring, often experience more obstetric complications and severe consequences compared to women without cancer [3,10,11,12,13]. Collectively, adverse pregnancy outcomes (APOs) are important contributors to maternal and neonatal morbidity and mortality [14,15]. Despite the well-established racial and ethnic disparities in cancer diagnosis, treatment, and comorbidities [16], little is known about the extent to which race and ethnicity influence APOs in women with cancer. Such data is essential to inform medical care and shared decision making between pregnant women with cancer and their providers [8,9]. Therefore, to address these gaps, the twofold aim of this study was to quantify the contemporary trends in the prevalence of cancer among singleton births by race and ethnicity in the United States, and to evaluate racial and ethnic disparities in the occurrence of APOs among cancer patients. Furthermore, we examine these effects by evaluating some of the common types of cancer reported to occur among pregnant women.

## 2. Materials and Methods

### 2.1. Study Population

Data for this retrospective cohort study are based on information from the National Inpatient Sample (NIS), which is the largest and most comprehensive all-payer inpatient care publicly available hospital database in the United States [17]. As part of the Healthcare Cost and Utilization Project (HCUP) and sponsored by the Agency for Healthcare Research and Quality, the NIS is designed to produce national estimates of inpatient utilization and outcomes for the United States [17]. Discharge information in NIS comprises one primary discharge diagnosis and up to 25 secondary diagnoses, as well as one primary procedure code and up to 15 secondary procedure codes [17]. Further details about the design and methodology of NIS are provided elsewhere [17]. The current study was limited to 17,632,568 singleton delivery-related hospital admissions (excluding induced abortions) among females (hereafter referred to as women) aged 18 to 49 years from 2000 to 2022. Because the NIS data is de-identified and publicly available, institutional review board approval was not required for the current study.

### 2.2. Measures

Patient-level maternal information for each hospitalization available in NIS includes age, sex, race and ethnicity, primary payer, median household income of patients’ zip code of residence, and length of stay. Race and ethnicity were defined by HCUP Partner organizations based on information provided by hospitals at the time of discharge. For the current study, the following categorizations were used: Non-Hispanic White, Non-Hispanic Black, Hispanic, Asian American and Pacific Islander, American Indian or Alaska Native, and other race/multiple races. Racial ethnic minority status was defined as all races and ethnicities besides non-Hispanic White. Hospital-level factors recorded were region, rural or urban location, and bed size. All clinical conditions, including pregnancies, cancer cases, APOs, and behavioral/lifestyle factors used for the current study were identified by querying from all diagnosis fields using the International Classification of Diseases (ICD) 9th and 10th clinical modification or procedure codes (Table S1). ICD-9 codes, which were available in NIS from 2000 to the third quarter of 2015, were translated to ICD-10-CM codes using publicly available conversion tools from the Centers for Medicare and Medicaid Services and a review of the literature on prior related research [18,19]. Factors considered in the current study include cancer, smoking/tobacco use, obesity, pre-existing hypertension and diabetes, and any depressive disorders. APO was defined as the presence of any of the following conditions on delivery discharge: hypertensive disorders of pregnancy, gestational diabetes, fetal growth restriction, intrauterine fetal demise and preterm birth. In-hospital maternal mortality was also evaluated. When missing data were present for the above-mentioned factors, they were coded as unknown. A comorbidity index was calculated based on up to 38 different pre-existing secondary conditions that are known to significantly influence in-hospital mortality and readmissions among adult populations using the Elixhauser Comorbidity Software (v2021.1) [20].

### 2.3. Statistical Analysis

The unit of analysis for the current study is delivery hospitalizations since NIS contains only discharge-level records, not patient-level records. Analyses were performed in accordance with recommendations by the Healthcare Cost and Utilization Project [21]. Discharge-level sampling weights accounting for the change in the design of the NIS in 2012 were used to generate national estimates. To estimate changes in national trends of the prevalence of cancer among delivery hospitalizations over time by race and ethnicity, Joinpoint regression was used to calculate average annual percent changes (AAPC). The test of the statistical significance of the trends over time was estimated using Monte Carlo Permutation methods [22]. Uncorrelated error models were constructed. To test the potential impact of the change in ICD codes on the estimated trends, comparability ratio models were implemented, which provide a direct estimation of trend data when there is a systematic change in data methodology, such as an ICD code change, and avoid bias associated with a standard joinpoint model in such cases [22]. A comparability ratio of 1.0093; thus, 1% more cases will be classified as having cancer when using ICD-10 than when using ICD-9 codes [23]. Survey-weighted means and percentages were calculated to describe women with cancer according to race and ethnicity, with comparisons made using analysis of variance for quantitative measures and the second-order Rao–Scott Chi-square tests for categorical measures. Logistic regression models incorporating the complex survey design were used to calculate odds ratios (OR) and 95% confidence intervals (CI) for the occurrence of APOs by race and ethnicity status among cancer hospitalizations. Factors adjusted in the multivariable models were maternal age, income, health insurance, region, rurality, hospital bed size, current smoker status, obesity and comorbidity index. These variables were considered as they are reported to either vary by race and ethnicity or are often associated with pregnancy outcomes [24,25]. To assess whether the association of race and ethnicity with APOs, as well as maternal mortality, differed among pregnant women with cancer compared to pregnant women without cancer, interaction tests were performed using the Wald Test. Owing to the type of conception (spontaneous conception or conception with assisted reproductive technology (ART) being associated with elevated risk for certain cancers as well as some APOs, a further interaction analysis was conducted between the type of conception and racial and ethnic groups on the risk of APO. ART data were only available for a subsample of the population as it first appeared in the NIS database in 2008. To evaluate the consistency of the estimated associations across subgroups of cancer (breast, cervix, ovary, thyroid, melanoma, hematologic, and all other cancers), subpopulation (domains) analyses were performed. Two-sided *p*-values < 0.05 were used to determine statistical significance. *p*-values were not adjusted for multiple testing as all analyses were prespecified. All statistical analyses were performed using the Joinpoint Regression Program, Version 5.4.0 (National Cancer Institute, Bethesda, MD, USA), SAS version 9.4 (SAS Institute Inc. Cary, NC, United States) and R software version 4.5.2 (R Foundation for Statistical Computing, Vienna, Austria).

## 3. Results

**Figure 1:**
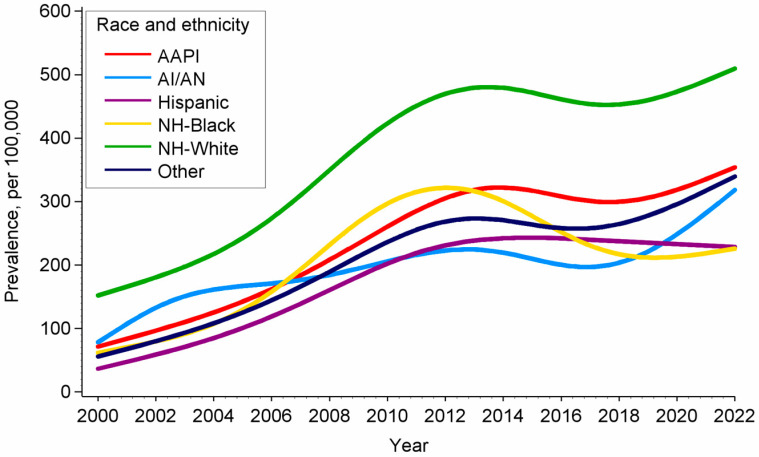
Temporal trends in the prevalence of cancer among pregnant women by race and ethnicity in the United States using Joinpoint regression analysis. 2000–2022. AAPI: Asian American and Pacific Islander; AI/AN: American Indian and Alaska Native. NH: Non-Hispanic.

From 2000 to 2022, the prevalence of cancer among singleton delivery hospitalizations increased by 225% from 120.4 per 100,000 to 391.8 per 100,000. Overall, the average annual percent change was 5.52 (95% CI: 3.60–7.48, *p* < 0.001) with the percent change greater for the period of 2000–2015 (AAPC = 7.68; 95% CI: 5.41–10.00) than 2016–2022 (AAPC = 3.37; 95% CI: 1.47–5.30). Despite fluctuations in the prevalence of cancer among pregnant women across race and ethnicity (Figure 1), there was a positive change in the AAPC among all race and ethnicity groups, although the estimate for American Indian and Alaska Native women did not approach statistical significance (*p* = 0.994). The average annual percent change in the prevalence of cancer over the 22-year period was highest for women of other or multiple races and ethnicities (AAPC = 8.26; 95% CI: 1.65–15.30) and lowest among non-Hispanic White women (AAPC = 5.49; 2.85–8.20). The AAPC for other racial and ethnic groups were as follows: Hispanic (AAPC = 7.85; 95% CI: 5.44–10.33); Asian American and Pacific Islander (AAPC = 7.50, 95% CI: 2.05–13.24), and non-Hispanic Black (AAPC = 5.59, 95% CI: 0.31–11.15).

Among the 17.6 million delivery hospitalizations, women with cancer had a higher mean (standard deviation) maternal age [33.4 (6.8) vs. 28.3 (5.8)] and a longer length of hospital stay [3.7 (5.1) vs. 2.6 (2.2) days]. A greater proportion of women with cancer used private insurance, were from urban locations, lived in high-income neighborhoods, were current smokers, had obesity, used ART, delivered via cesarean section and had pre-pregnancy conditions like hypertension and diabetes compared to women without cancer (Table S2). Furthermore, women with cancer had a greater prevalence of any APOs (28.1% vs. 22.0%). Characteristics of the 49,824 delivery hospitalizations among women with cancer are presented in Table 1.

**Figure 2:**
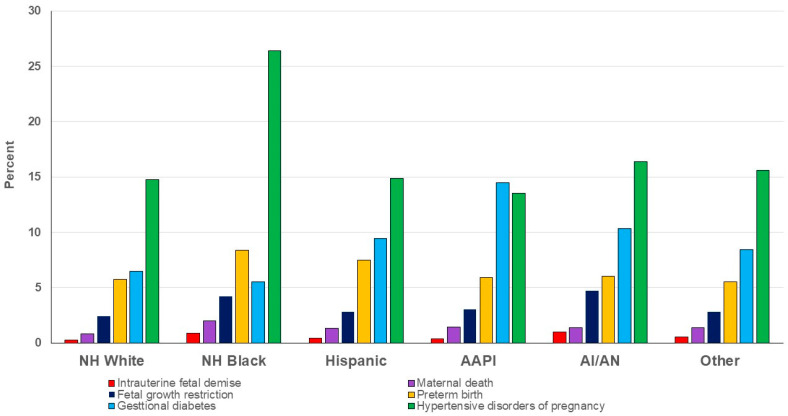
Prevalence of adverse pregnancy outcomes among deliveries to mothers with cancer. AAPI: Asian American or Pacific Islander, AI/AN: American Indian and Alaska Native, NH: Non-Hispanic.

Overall, Asian American and Pacific Islander and non-Hispanic Black women had the highest maternal age. A greater proportion of Hispanic, American Indian and Alaska Native, and non-Hispanic Black women lived in locations with lower median household income and used Medicaid as a primary form of payment. Both American Indian and Alaska Native and non-Hispanic Black women had the highest prevalence for obesity, current tobacco use, and pre-existing health conditions such as hypertension and diabetes. The prevalence of any APOs was highest among American Indian and Alaska Native (33.8%) and non-Hispanic Black (39.2%) women, while being lowest among non-Hispanic White women (26.3%) (Figure 2).

**Figure 3:**
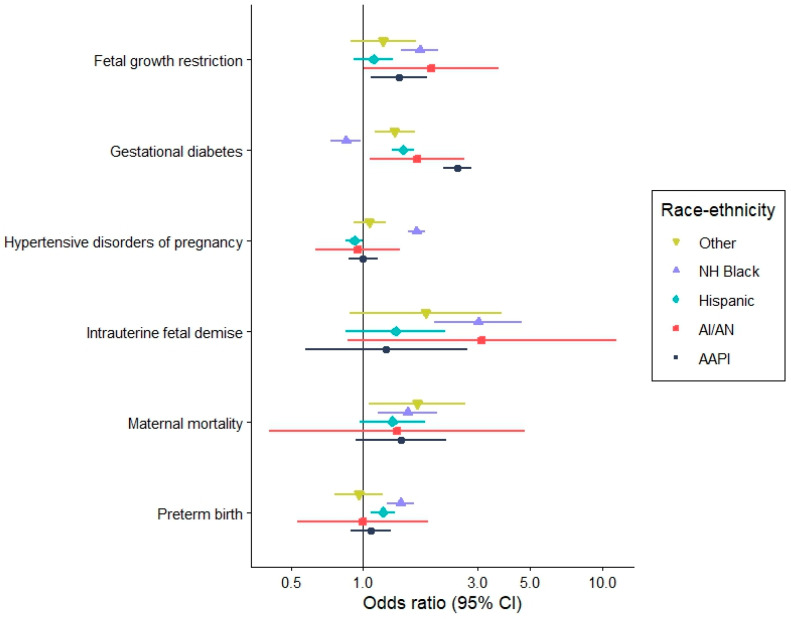
Adverse pregnancy outcomes among women with cancer by race and ethnicity with NH White as referent. AAPI: Asian American or Pacific Islander, AI/AN: American Indian and Alaska Native, CI: confidence interval, NH: Non-Hispanic.

After accounting for demographic, socioeconomic, behavioral/lifestyle factors and comorbidity index among women with cancer, the occurrence of APOs tended to be higher among racial and ethnic minority women compared to non-Hispanic White women (Figure 3, Table S3). For instance, non-Hispanic Black women had the highest odds for hypertensive disorders of pregnancy (OR = 1.67, CI: 1.54–1.82), preterm birth (OR = 1.44, CI: 1.26–1.64) and intrauterine fetal demise (OR = 3.04, CI: 1.99–4.63). Asian American and Pacific Islander women had the highest odds for gestational diabetes (OR = 2.48, CI: 2.17–2.85), American Indian and Alaska Native women had the highest odds for fetal growth restriction (OR = 1.92, CI: 1.00–3.69), while women of other/multiple races had the highest odds for maternal mortality (OR = 1.72, CI: 1.08–2.75).

**Figure 4:**
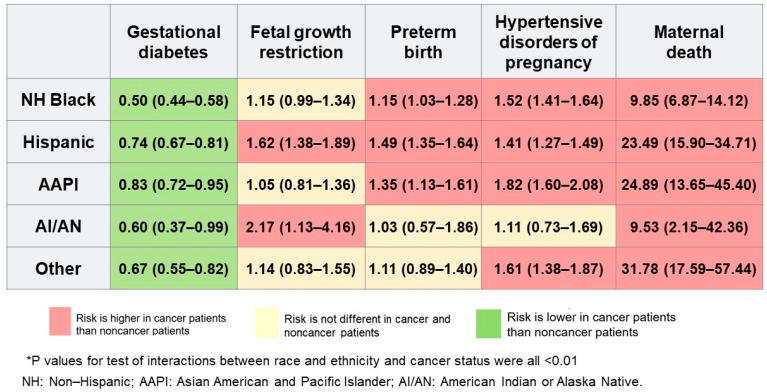
Odds ratios and 95% confidence intervals for the comparison of estimates for adverse pregnancy outcomes between women with and without cancer by race and ethnicity, with non-Hispanic White women as referent.

There was no significant interaction between ART use and race and ethnicity on the risk of APOs (*p* = 0.454). Interaction tests evaluating whether the association of race and ethnicity with APOs, as well as maternal mortality, differed among pregnant women with cancer compared to pregnant women without cancer were all statistically significant (*p* < 0.01) with the exception of intrauterine fetal demise (*p* = 0.589). Comparing estimates for women with and without cancer, where non-Hispanic White women were referent, the odds for maternal mortality were significantly higher among women with cancer for all racial/ethnic minority groups as well as several of the APOs (Figure 4). Of note, the odds for maternal mortality among racial and ethnic minority women ranged from 9-fold to 32-fold. Conversely, all racial/ethnic minority groups with cancer had significantly lower odds for gestational diabetes compared to women without cancer.

**Figure 5:**
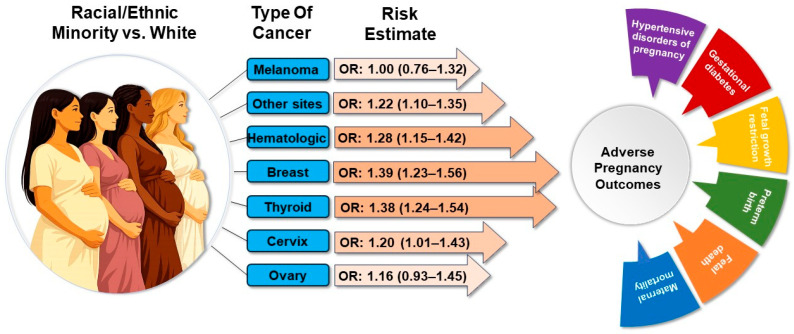
Adverse pregnancy outcomes according to type of cancer, comparing racial/ethnic minority women to non-Hispanic White women. Effect estimates adjusted for age, income, health insurance, rurality, region, hospital bed size, smoking status, obesity and comorbidity index. OR: odds ratio.

Characteristics of the delivery hospitalizations among women with cancer according to the site of cancer are presented in Table S4. The proportions of the types of cancer evaluated are as follows: hematologic (19.9%), thyroid (18.3%), breast (13.9%), melanoma (11.7%), cervix (8.7%) and ovary (4.7%). With regard to the type of cancer, compared to non-Hispanic White women, the risk of any APO (including maternal mortality) among racial/ethnic minority women was highest for breast (OR = 1.39, CI: 1.23–1.56), thyroid (OR = 1.38, CI: 1.24–1.54), and hematologic (OR = 1.28, CI: 1.15–1.42) cancers, with these association often limited to non-Hispanic Black women (Figure 5 and Table S5).

## 4. Discussion

In this large population-based study spanning 22 years, the prevalence of early onset cancer among singleton delivery hospitalizations increased by 225%. While this increasing trend was observed among all races and ethnicities, the greatest changes occurred among racial and ethnic minority groups. Additionally, significant racial and ethnic disparities in APOs and maternal mortality were observed among women with concurrent early onset cancer diagnosis at delivery. Targeted management of APO risk factors during pregnancy among racial/ethnic minority populations with cancer may help reduce adverse maternal and neonatal outcomes.

These findings, which provide contemporary data that confirm an increasing trend in the prevalence of cancer among delivery hospitalizations, also extend prior work conducted in the United States and other European countries. Thus, this current analysis includes information for all racial and ethnic minority groups represented in the United States who often encounter a greater burden of health conditions [3,4]. Reasons for this increasing trend are not completely known but may be related to an increasing screening or ascertainment of cancer diagnoses that may influence the rising prevalence of cancer among reproductive age women [3,6], increasing maternal age at birth [26], and improved treatment modalities that enhance cancer survival and fertility preservation [27,28,29]. The particularly high increase in the prevalence of cancer during delivery hospitalization among racial ethnic minority women, such as Hispanic women who are the second largest racial/ethnic group in the United States, is reflective of the well-documented racial/ethnic disparities in the incidence and outcomes of cancer in the United States [16]. Such disparities are often driven by a combination of structural, socioeconomic, and behavioral factors, as well as genetic and hereditary factors that interact with environmental factors to adversely affect racial and ethnic minority groups [30].

The increasing trend in the prevalence of cancer during delivery poses important challenges in obstetric and oncologic care and management of this growing population and their offspring [8,9,10,11,12]. Up until now, it was unknown how these challenges affected racial and ethnic minority women who are known to have a greater burden of APOs in the absence of cancer [31]. The observation in the current study of significant racial and ethnic disparities in APOs and maternal mortality among women with cancer, especially non-Hispanic Black women, which was consistent across several types of cancers like breast, cervical, hematologic and thyroid, consistently follows patterns observed in non-cancer populations [31,32]. However, it is worth noting that in the current study, racial ethnic minority women with cancer, to a large extent, had a higher risk of several APOs and maternal mortality, ranging from 9-fold to 32-fold, compared to racial ethnic minority women without cancer. Specifically, hypertensive disorders of pregnancy and preterm births were all higher among non-Hispanic Black, Hispanic and Asian American and Pacific Islander women, while fetal growth restriction was higher among Hispanic and American Indian and Alaska Native women compared to the corresponding racial and ethnic minority group without cancer.

Another interesting finding from the current study is that although women with cancer had better socioeconomic status than women without cancer, they were older at the time of birth and had worse health profiles before the onset of pregnancy. This observation seems to contradict findings from studies mainly among women without cancer, where high socioeconomic status was observed to predispose to better pregnancy outcomes beyond underlying pre-pregnancy health status [33]. An individual’s lifestyle is often shaped by their socioeconomic status, and a high socioeconomic status provides an opportunity for adequate resources to maintain a healthy lifestyle [34]. Accordingly, some studies report that the influence of socioeconomic status on health outcomes is mediated by certain health-promoting behaviors and lifestyles [34]. Generally, among women with cancer, those with high socioeconomic status often have better outcomes [35]. However, among women with high socioeconomic status, macro-social factors and adoption of unhealthy lifestyles, such as high-calorie diet intake and low physical activity, which are known risk factors for cancer, coupled with chronic stress, can contribute to poor health before the onset of cancer [34]. Even at high socioeconomic status, cancer disparities exist, with racial and ethnic minority women reported to not achieve equivalent gains in cancer health as people of White race [35,36].

The drivers of racial and ethnic disparities in APOs among women with cancer are complex and multifactorial; such observation suggests that better socioeconomic factors do not necessarily counteract the effects of chronic physiological stress experienced over the life course by some racial and ethnic minority groups with cancer [32,37,38,39]. Accordingly, chronic psychological stress is reported to accelerate telomere shortening and cellular aging, which in turn influence the development of chronic diseases and tumorigenesis [32,40]. These pre-pregnancy chronic conditions tend to be more prevalent and less well controlled among some subgroups of racial and ethnic minority groups, which increases their risk for APOs [41,42]. Thus, in the context of adverse outcomes during pregnancy, the findings of the current study highlight the additional vulnerability that the interaction of individual behaviors and biological or genetic factors, which often have roots in health inequalities, and are compounded by unfavorable environmental factors, poses to racial and ethnic minority women with cancer [30,32,43]. There remains an unmet need to understand the racial and ethnic disparities in APOs, especially among women with cancer.

Although the role of cancer treatment modality was not evaluated in the current study, several lines of evidence show that some cancer therapeutics are associated with adverse outcomes, including stillbirth, preterm birth and fetal growth restriction, while the evidence for the association between cancer treatment and APOs, like hypertensive disorders of pregnancy and gestational diabetes, remains equivocal [15,44]. Unlike surgery and chemotherapy, radiation therapy is rarely used in the early stages of conception, especially when it is being applied to the ovaries, uterus, and pituitary gland to avoid miscarriages and structural or functional anomalies in the fetus [15,45]. Accordingly, radiation therapy or in combination with chemotherapy has been reported to be associated with elevated risk for gestational diabetes, small for gestational age and low birth weight [46]. When used in the first trimester, chemotherapy is associated with embryonic and fetal toxicity as well as congenital malformations and spontaneous miscarriage, while preterm delivery and low birth weight are common effects of chemotherapy, including immunosuppression, when initiated after the first trimester [47,48,49,50,51]. Although some reports often attribute APOs in relation to chemotherapy or radiation to short intervals between treatment and conception, others show that these treatment modalities may have enduring effect on pregnancy outcomes several years after treatment completion [13,52,53], with proposed mechanisms often including gonadal damage, uterine vascular impairment, and hypothalamic–pituitary axis dysfunction as a result of these therapies [15,54]. The implications of race and ethnicity in these findings are unclear. However, racial and ethnic disparities in cancer treatment are well documented. Women from minority racial and ethnic groups are less likely to receive guideline-concordant care [55] and also decline recommended primary cancer treatment modalities consisting of chemotherapy and radiotherapy [56,57]. Therefore, it is especially important that future studies on APOs among women with cancer consider the unique roles of these treatment-related disparities in light of the widely reported racial and ethnic differences in access to healthcare, sociodemographic, socioeconomic, behavior and lifestyle, and clinical factors that are known to influence cancer outcomes.

In all, the findings of the current study suggest that among pregnant women with cancer, risk stratification by race and ethnicity should be considered with screening algorithms implemented for adverse pregnancy conditions like hypertensive disorders of pregnancy, gestational diabetes and fetal growth restriction. Also, investigations are needed to determine whether the treatment and management of cancer in women during pregnancy should be similar to those provided to non-pregnant women.

The strengths of the current study include the use of a nationally representative sample from the largest and extensively validated all-payer inpatient database in the United States, which enhances the generalizability of the findings. As with any retrospective cohort study using healthcare administrative data, the following limitations should be considered. Pregnancy losses that did not result in hospitalizations, out-of-hospital deliveries, and postpartum information beyond the delivery admission were not captured in the NIS database, which could result in underestimation of the reported trends and associations. Furthermore, the prevalence of certain factors, like ART use, is underestimated in administrative data like NIS as a result of incomplete discharge documentation. Due to the newborn data in NIS not being linked to maternal discharge information, APOs like neonatal intensive care unit hospitalization and other neonatal-related conditions were not evaluated. Also, information on clinical events such as cancer treatment, medication use and laboratory results, as well as data on events like date of conception, date of cancer diagnosis, and date of hospitalization beyond the year of hospitalization, was not available in the NIS database. Cancer, APOs and other clinical conditions were defined using ICD codes, which are prone to error during the coding process. Related to that, the use of secondary diagnosis codes for defining these conditions also made it impossible to distinguish between pregnancy among cancer survivors and pregnancy-associated cancer. However, with 71% of pregnancy-associated cancer diagnosed during the 1-year postpartum period, and 33% of pregnancy-associated cancer diagnosed during pregnancy resulting in induced abortions [58], the possibility of this condition influencing the reported outcomes is minimal as hospitalization for induced abortions was excluded from the current analysis. Finally, although several potential confounders were accounted for in the models, residual confounding cannot be entirely ruled out.

## 5. Conclusions

This study among women with early onset cancer, together with several others among women without cancer, informs efforts to achieve maternal health equity in the United States. Specifically, it shows an increasing trend in the prevalence of early onset cancer among singleton delivery hospitalizations and makes a novel contribution by showing significant racial and ethnic disparities in APOs among women with cancer. For several APOs, this disparity among women with cancer is significantly higher than that reported among women without cancer. Despite accounting for individual-level and neighborhood-level demographic and socioeconomic factors, behavior and lifestyle factors and a large array of comorbid chronic diseases, racial and ethnic disparities in APO persisted among women with cancer, suggesting that other extrinsic factors related to the environment, health facility, provider, and healthcare systems may potentially drive the racial and ethnic disparities observed among women with cancer. With racial and ethnic groups often not being monolithic groups, further research is needed to better understand racial and ethnic disparities in APOs among women with cancer, and how best obstetricians and oncologists can manage this vulnerable population of women with concurrent cancer and pregnancy, especially those from racial and ethnic minority backgrounds.

## Figures and Tables

**Table 1:** Characteristics of pregnant women with early onset cancer according to race and ethnicity, NIS 2000–2022.

Characteristics ^a^	Race and Ethnicity						*p* Value ^b^
	NH White (n = 30,103)	NH Black (n = 4517)	Hispanic (n = 5851)	AAPI (n = 2053)	AI/AN (n = 213)	Other (n = 1499)	
Age, years	33.4 (6.4)	34.2 (8.1)	32.9 (7.3)	35.1 (5.8)	32.9 (7.7)	33.9 (6.6)	0.038
Median household income, %							<0.001
0–25th percentile	4478 (14.9)	1887 (41.8)	1699 (29.0)	174 (8.5)	67 (30.7)	233 (15.7)	
26th–50th percentile	6336 (21.0)	933 (20.8)	1434 (24.5)	290 (14.1)	64 (30.3)	288 (19.0)	
51st–75th percentile	8130 (27.0)	886 (19.6)	1506 (25.7)	483 (23.6)	36 (17.0)	383 (25.4)	
76th–100th percentile	10,843 (36.0)	711 (15.7)	1118 (19.2)	1079 (52.5)	33 (15.8)	540 (36.2)	
Unknown	311 (1.0)	98 (2.1)	94 (1.6)	27 (1.3)	13 (6.1)	55 (3.7)	
Primary payer, %							<0.001
Medicaid	6596 (21.9)	2414 (53.5)	2908 (49.6)	405 (19.8)	98 (45.7)	496 (33.2)	
Private	22,073 (73.3)	1809 (40.0)	2462 (42.2)	1545 (75.2)	96 (45.2)	891 (59.4)	
Other	1400 (4.6)	292 (6.5)	475 (8.1)	101 (4.9)	19 (9.2)	111 (7.4)	
Hospital region, %							<0.001
Northeast	7260 (24.1)	896 (19.9)	918 (15.8)	456 (22.2)	22 (10.4)	521 (34.8)	
Midwest	6694 (22.3)	789 (17.6)	452 (7.8)	195 (9.6)	39 (18.6)	238 (15.7)	
South	9816 (32.6)	2402 (53.0)	1990 (33.9)	370 (18.0)	68 (31.0)	486 (32.5)	
West	6333 (21.0)	430 (9.6)	2491 (42.6)	1032 (50.2)	84 (40.0)	254 (16.9)	
Location, rural areas, %	3930 (13.1)	320 (7.0)	263 (4.6)	50 (2.4)	65 (29.9)	102 (6.7)	<0.001
Clinical history, %							
Smoker or tobacco use	3193 (10.6)	518 (11.5)	231 (3.9)	54 (2.7)	23 (10.9)	85 (5.8)	<0.001
Obesity	2542 (8.5)	633 (14.1)	710 (12.3)	94 (4.7)	30 (14.5)	125 (8.4)	<0.001
Pre-existing hypertension	1670 (5.6)	749 (16.6)	373 (6.3)	123 (6.0)	22 (10.2)	99 (6.8)	<0.001
Pre-existing diabetes	652 (2.2)	251 (5.5)	246 (4.2)	56 (2.8)	18 (8.5)	48 (3.4)	<0.001
Depressive disorders	2349 (7.8)	321 (7.1)	309 (5.3)	60 (3.0)	15 (7.3)	68 (4.5)	<0.001
Cesarian section	9780 (32.5)	1294 (28.7)	1948 (33.3)	649 (31.5)	65 (30.4)	538 (35.9)	<0.001
Any APOs	7898 (26.3)	1764 (39.2)	1774 (30.3)	650 (31.8)	72 (33.8)	430 (28.9)	<0.001
Length of hospital stay, days	3.4 (4.4)	5.0 (7.2)	4.2 (6.1)	3.9 (4.7)	4.0 (4.1)	4.0 (6.1)	<0.001

AAPI: Asian American or Pacific Islander, AI/AN: American Indian and Alaska Native, NH: Non-Hispanic. ^a^ Values are weighted means (and standard deviation) for continuous variables, and unweighted frequencies and weighted percentages for categorical variables. ^b^ Based on the Rao–Scott chi-square test for categorical variables and survey-weighted linear regression for continuous variables.

## Data Availability

The National Inpatient Sample data used in this analysis are publicly available from the Hospital Cost and Utilization Project website (https://hcup-us.ahrq.gov/tech_assist/centdist.jsp (Accessed on 9 February 2026). Access to the data is subject to the Hospital Cost and Utilization Project data use agreements.

## Supplementary Materials

The following supporting information can be downloaded at: https://www.mdpi.com/article/10.3390/cancers18071081/s1. Table S1: International classification of diseases (ICD) diagnoses and procedure codes for defining diseases or conditions; Table S2: Characteristics of pregnant women according to early onset cancer status, NIS 2000–2022; Table S3: Odds ratios and 95% confidence intervals for adverse pregnancy outcomes among women with early onset cancer according to race and ethnicity; Table S4: Characteristics of pregnant women with early onset cancer according to type of cancer, NIS 2000–2022; Table S5: Odds ratios and 95% confidence intervals for adverse pregnancy outcomes among women with early onset cancer according to race and and cancer site.

## Abbreviations

The following abbreviations are used in this manuscript:

AAPC	Average annual percent change
ART	Assisted reproductive technology
AAPI	Asian American or Pacific Islander
AI/AN	American Indian and Alaska Native
APO	Adverse pregnancy outcome
CI	Confidence interval
HCUP	Healthcare Cost and Utilization Project
NH	Non-Hispanic
NIS	National Inpatient Sample
ICD	International classification of diseases
OR	Odds ratios
