# Supplementary material for: Racial and Ethnic Disparities in Adverse Pregnancy Outcomes Among Women with Early Onset Cancer in the United States
Source: Cancers (Basel). 2026 Mar 26;18(7):1081. doi: 10.3390/cancers18071081 (PMC13072104; doi:10.3390/cancers18071081)
Supplement: Supplementary file 1 [file cancers-18-01081-s001.zip › cancers-4188325-supplementary.pdf]

SUPPLEMENTARY MATERIALS

Racial and Ethnic Disparities in Adverse Pregnancy Outcomes Among women with Early-onset Cancer in the United States

Duke Appiah, PhD, MPH, MS; Julie Sang, MPH; Eric K Broni, MD and MPH; Zheng Shi, MD, PhD; Catherine Kim, MD and MPH

Table S1. International classification of diseases (ICD) diagnoses and procedure codes for defining diseases or conditions.

Disease or condition	ICD-9	ICD-10
Delivery hospitalization	V27.x, 650.x, procedure code: 72.0-75.9, DRG (Diagnosis Related Group) codes: 370, 371, 372, 373, 374, 375, 765, 766, 767, 768	O60-O77.x, O80.x, O82.x, Z37.x, Z38.x, procedure codes: 10D00Z0- 10D00Z8, 10E0XZZ
Cesaerean section	669.7, procedure code: 74.0-74.9	O82.x, P072, P073, procedure code: 10D00Z0, 10D00Z1, 10D00Z2
Induced abortion	635.x, 636.x, 637.x, 638.x	O04, Z33.2, procedure code: 10A00ZZ, 10A03ZZ, 10A04ZZ, 10A07Z6, 10A07ZW, 10A07ZX, 10A07ZZ, 10A08ZZ
Multiple births	V30.x-V39.x, 651.0–651.9, 652.6, 659.4, V27.2-V27.7	Z37.2- Z37.7, O30.x
Assisted reproductive technology	V2385	O0981x
Hypertensive disorders of pregnancy	642.0-642.9, 401-405.x	O10-O11.x, O13-O16.x, I10-I13.x, I15.x
Gestational diabetes	648.8x.	O24.4x
Fetal growth restriction	656.5x, 764.9	O36.4, O36.5, Z36.4
Intrauterine fetal demise	656.4, 768.1	O36.4
Preterm birth	765.1, 765.2, 644.21	O60.1, P07.2, P07.3
Prevalent hypertension	401-405.x, 642.0-642.2, 642.7	O10.x, I10.x, I15.x
Prevalent diabetes	648.0, 250.x,	O24.0, O24.1, O24.3, O24.8, E10.x, E11.x, E13.x, E14.x
Depressive disorders	296.20-296.24, 296.30-296.34, 300.4x, 311.x,	F32.x, F33.x, F34.1, F43.21, F43.23
Smoker or tobacco user	305.1, 649.0, V15.82	F17.x, O99.33
Obesity	278.0, 649.1, V85.3, V85.4	O99.21, Z683, Z68.39, Z68.4, Z68.45, E66.x
Cancer	140–239.x	C00-C96.x, O9A1, Z85.42, Z85.9
Breast cancer	174.x, V103	C50.x, Z853
Cervical cancer	180.x, V1041	C53.x, Z85.41
Ovarian cancer	183.x, V1043	C56-57.x, Z85.43
Melanoma	172.x, V1082	C43.x, Z85.820
Thyroid cancer	193.x, V1087	C73.x, Z85.850
Hematologic cancer	200-208.x, V106-107	C81-93.x, Z85.6, Z857

Table S2. Characteristics of pregnant women according to early onset cancer status, NIS 2000-2022.

	Cancer status		
Characteristics [a]	No (n=17,582,744)	Yes (n=49,824)	P value [b]
Age, years	28.3 (5.8)	33.4 (6.8)	<0.001

Race and ethnicity, %			<0.001
Non-Hispanic White	45.3	60.6	
Non-Hispanic Black	11.9	9.1	
Hispanic	19.1	11.8	
Asian or Pacific Islander	4.7	4.1	
American Indian and Alaska Native	0.6	0.4	
Other	4.0	3.0	
Unknown	14.3	10.9	
Median household income, %			<0.001
0–25th percentile	24.1	18.8	
26th–50th percentile	24.2	21.3	
51st–75th percentile	24.4	25.9	
76th–100th percentile	25.8	32.6	
Unknown	1.4	1.3	
Primary payer, %			<0.001
Medicaid	41.3	28.8	
Private	52.7	65.7	
Other	5.8	5.4	
Unknown	0.2	0.1	
Hospital region, %			<0.001
Northeast	16.3	20.7	
Midwest	21.1	22.9	
South	38.4	32.8	
West	24.3	23.6	
Location, %			<0.001
Urban	85.3	87.7	
Rural	14.0	11.4	
Unknown	0.7	0.9	
Clinical history, %			
Smoker or tobacco use	5.3	9.3	<0.001
Obesity	5.9	8.9	<0.001
Pre-existing hypertension	2.3	6.7	<0.001
Pre-existing diabetes	1.1	2.8	<0.001
Depressive disorders	2.4	6.9	<0.001
Assisted reproductive technology	0.1	0.5	<0.001
Cesarian section	30.3	32.3	<0.001
Any APOs	22.0	28.1	<0.001
Length of hospital stay, days	2.6 (2.2)	3.7 (5.1)	<0.001

[a] Values are weighted means (and standard deviation) for continuous variables, and percentages for categorical variables. [b] Based on Rao-Scott chi-square test for categorical variables and survey-weighted linear regression for continuous variable.

Table S3. Odds ratios and 95% confidence intervals for adverse pregnancy outcomes among women with early onset cancer according to race and ethnicity.

Adverse pregnancy outcomes	Non-Hispanic White	Non-Hispanic Black	Hispanic	Asian American or Pacific Islander	American Indian and Alaska Native	Other
	OR (95% CI)	OR (95% CI)	OR (95% CI)	OR (95% CI)	OR (95% CI)	OR (95% CI)
Hypertensive disorders of Pregnancy	1	1.67 (1.54-1.82)	0.92 (0.84-1.00)	1.00 (0.87-1.15)	0.95 (0.64-1.43)	1.07 (0.92-1.24)
Gestational diabetes	1	0.85 (0.73-0.98)	1.47 (1.32-1.63)	2.48 (2.17-2.85)	1.68 (1.07-2.64)	1.36 (1.12-1.65)
Fetal growth restriction	1	1.73 (1.45-2.07)	1.10 (0.91-1.33)	1.42 (1.08-1.86)	1.92 (1.00-3.69)	1.22 (0.89-1.67)

Intrauterine fetal demise	1	3.04 (1.99-4.63)	1.37 (0.85-2.21)	1.25 (0.57-2.72)	3.13 (0.86-11.44)	1.83 (0.88-3.80)
Preterm birth	1	1.44 (1.26-1.64)	1.21 (1.07-1.36)	1.07 (0.88-1.31)	0.99 (0.53-1.87)	0.96 (0.76-1.21)
Maternal mortality	1	1.54 (1.15-2.05)	1.33 (0.97-1.81)	1.44 (0.93-2.23)	1.38 (0.40-4.73)	1.69 (1.06-2.69)

CI: confidence interval, OR: odds ratio. Model adjusted for age, income, health insurance, rurality, region, hospital bed size, smoking status, obesity and comorbidity index.

Table S4. Characteristics of pregnant women with early onset cancer according to type of cancer, NIS 2000-2022.

	**Race and ethnicity**							
Characteristics [a]	Breast (n= 6877)	Cervix (n= 4319)	Hematologic (n=9841)	Melanoma (n= 5758)	Ovary (n= 2334)	Thyroid (n=9006)	All other sites (n=10759)	P value [b]
Age, years	36.0 (5.6)	32.3 (6.2)	30.2 (6.0)	32.7 (4.9)	34.3 (8.1)	32.4 (5.2)	35.8 (8.1)	<0.001
Race and ethnicity, %								<0.001
Non-Hispanic White	54.2	63.6	58.6	84.0	52.9	60.8	54.2	
Non-Hispanic Black	14.4	8.9	10.4	0.6	11.0	4.2	12.7	
Hispanic	11.2	10.6	13.0	2.7	16.5	13.9	13.6	
Asian or Pacific Islander	6.4	2.3	2.7	0.6	5.5	7.0	4.0	
American Indian and Alaska Native	0.4	0.7	0.4	0.2	0.3	0.3	0.6	
Other	3.7	2.2	3.0	1.5	3.6	3.8	3.0	
Unknown	9.8	11.8	11.9	10.4	10.2	9.9	12.1	
Median household income, %								<0.001
0–25th percentile	17.4	23.0	20.8	10.0	22.1	14.5	23.7	
26th–50th percentile	19.3	23.5	22.6	18.7	23.2	20.7	22.2	
51st–75th percentile	24.9	25.9	25.4	28.1	25.2	27.4	24.6	
76th–100th percentile	36.9	25.9	29.9	42.6	28.2	36.5	27.8	
Unknown	1.6	1.7	1.2	0.7	1.3	0.9	1.8	
Primary payer, %								<0.001
Medicaid	25.6	42.5	33.2	11.3	36.4	21.4	35.0	
Private	69.4	51.4	62.3	84.6	57.0	74.1	57.0	
Other	4.9	6.0	4.4	4.0	6.5	4.2	7.9	
Unknown	0.1	0.1	0.1	0.1	0.0	0.2	0.2	
Hospital region, %								<0.001
Northeast	21.6	15.5	21.3	20.5	20.0	23.7	19.7	
Midwest	20.1	27.0	23.2	25.7	22.1	22.5	21.9	
South	34.9	34.7	33.4	30.9	31.3	28.0	35.4	
West	23.4	22.8	22.2	22.9	26.6	25.7	23.1	
Location, rural areas, %	9.2	13.7	12.7	10.0	11.8	10.0	12.5	<0.001
Clinical history, %								
Smoker or tobacco use	6.4	21.7	6.9	5.8	11.1	4.2	14.0	<0.001
Obesity	7.1	8.8	10.0	7.6	11.3	10.8	6.9	<0.001
Pre-existing hypertension	5.8	5.7	4.8	3.3	9.2	4.4	11.8	<0.001
Pre-existing diabetes	2.4	2.6	2.0	1.0	4.3	2.4	4.6	<0.001
Depressive disorders	6.1	7.6	6.2	6.2	8.1	5.3	8.8	<0.001
Cesarian section	35.7	36.6	35.0	34.3	31.6	34.5	23.5	<0.001

Any APOs	29.6	28.4	29.2	23.5	28.1	27.3	28.2	<0.001
Length of hospital stay, days	3.5 (4.5)	3.5 (4.3)	3.5 (5.3)	2.7 (2.4)	4.5 (5.6)	2.8 (2.6)	5.2 (7.2)	<0.001

AAPI: Asian American or Pacific Islander, AI/AN: American Indian and Alaska Native CI: confidence interval, NH: Non-Hispanic.[a] Values are weighted means (and standard deviation) for continuous variables, and percentages for categorical variables. [b] Based on Rao-Scott chi-square test for categorical variables and survey-weighted linear regression for continuous variables.

Table S5. Odds ratios and 95% confidence intervals for adverse pregnancy outcomes among women with early onset cancer according to race and ethnicity and cancer site.

**Cancer site**	**Non-Hispanic White**	**Non-Hispanic Black**	**Hispanic**	**Asian American or Pacific Islander**	**American Indian and Alaska Native**	**Other**	**P value**
	OR (95% CI)	OR (95% CI)	OR (95% CI)	OR (95% CI)	OR (95% CI)	OR (95% CI)	
Breast	1	1.57 (1.33-1.84)	1.20 (1.01-1.43)	1.37 (1.10-1.71)	1.22 (0.58-2.58)	1.32 (1.00-1.74)	<0.001
Cervix	1	1.41 (1.11-1.79)	1.14 (0.89-1.46)	1.27 (0.80-2.01)	1.25 (0.59-2.64)	0.71 (0.43-1.18)	0.059
Ovary	1	1.24 (0.91-1.71)	1.05 (0.78-1.40)	1.38 (0.91-2.09)	1.04 (0.19-5.70)	1.03 (0.62-1.71)	0.375
Thyroid	1	1.87 (1.50-2.33)	1.19 (1.02-1.38)	1.57 (1.32-1.87)	1.38 (0.58-3.25)	1.18 (0.92-1.52)	<0.001
Melanoma	1	1.94 (0.94-4.01)	0.92 (0.68-1.36)	0.94 (0.40-2.22)	1.52 (0.48-4.79)	0.81 (0.48-1.36)	0.380
Hematologic	1	1.44 (1.23-1.68)	1.18 (1.02-1.36)	1.36 (1.05-1.76)	0.97 (0.46-2.03)	1.20 (0.93-1.54)	<0.001
All other sites	1	1.57 (1.36-1.80)	0.96 (0.83-1.10)	1.14 (0.90-1.46)	1.42 (0.79-2.56)	1.07 (0.81-1.42)	<0.001

CI: confidence interval, OR: odds ratio. Model adjusted for age, income, health insurance, rurality, region, hospital bed size, smoking status, obesity and comorbidity index.
